# Comparing indicators of disease severity among patients presenting to hospital for urinary tract infections before and during the COVID-19 pandemic

**DOI:** 10.1093/jacamr/dlae067

**Published:** 2024-04-24

**Authors:** Selina Patel, Martin Gill, Andrew Hayward, Susan Hopkins, Andrew Copas, Laura Shallcross

**Affiliations:** Institute of Health Informatics, University College London, London, UK; UK Health Security Agency, London, UK; University Hospitals Birmingham NHS Foundation Trust, Birmingham, UK; Institute of Health Informatics, University College London, London, UK; UK Health Security Agency, London, UK; UK Health Security Agency, London, UK; Division of Infection and Immunity, University College London, London, UK; Institute for Global Health, University College London, London, UK; Institute of Health Informatics, University College London, London, UK; UK Health Security Agency, London, UK

## Abstract

**Background:**

During the COVID-19 pandemic, patients may have delayed seeking healthcare for urinary tract infections (UTIs). This could have resulted in more severe presentation to hospital and different antibiotic usage.

**Objectives:**

We explored evidence for such changes through existing national indicators of prescribing, and routine clinical data collected in the electronic health record (EHR).

**Methods:**

We carried out a retrospective cohort study of patients presenting to two UK hospitals for UTIs, comparing two indicators of disease severity on admission before and during the pandemic: intravenous (IV) antibiotic use, and National Early Warning Score 2 (NEWS2). We developed regression models to estimate the effect of the pandemic on each outcome, adjusting for age, sex, ethnicity and index of multiple deprivation.

**Results:**

During the pandemic, patients were less likely to present to hospital for UTI with NEWS2 of 0 or 1 [adjusted odds ratio (aOR): 0.66; 95% confidence interval (CI): 0.52–0.85] compared with before, more likely to present with score 2 (aOR: 1.52; 95% CI: 1.18–1.94), whereas the likelihood of presenting with a NEWS2 of >2 remained the same (aOR: 1.06; 95% CI: 0.87–1.29). We did not find evidence that this limited increase in disease severity resulted in changes to IV antibiotic use on admission (adjusted risk ratio: 1.02; 95% CI: 0.91–1.15).

**Conclusions:**

There may have been a small increase in disease severity at hospital presentation for UTI during the pandemic, which can be detected using routine data and not through national indicators of prescribing. Further research is required to validate these findings and understand whether routine data could support a more nuanced understanding of local antimicrobial prescribing practices.

## Introduction

During the first 2 years of the COVID-19 pandemic in England, National Health Service (NHS) Trusts experienced changes to patient populations as they provided care through three waves of infection.^1^ In 2020–2021, Emergency Department (ED) attendances fell by 30% compared with 2019–2020, although this reduction varied with socioeconomic deprivation, and attendances in the most deprived decile of the population remained double those in the least deprived population decile.^2^ The majority of the decline was observed in lower acuity attendances.^3^ However, there are reports of these changes in hospital presentation being driven in part by patients delaying seeking healthcare, resulting in later stage and more severe eventual presentation to hospital.^4^ For patients seeking care for infections, this behaviour may have resulted in changes to antimicrobial usage on admission to hospital, potentially driving the emergence and spread of antimicrobial drug resistance.

Whereas total antibiotic consumption in NHS hospitals fell during the first year of the pandemic, the rate of use per admission increased by 4.8%.^5^ In primary care, consultations for infection fell along with antibiotic consumption.^6^ Social restrictions interrupted exposure and transmission of common infections, particularly those in the upper and lower respiratory tract. However, there were also reports of a small decline in rates of patients seeking care for infections expected to be largely unaffected by the pandemic, such as urinary tract infections (UTIs).^7,8^ The underlying epidemiology of UTIs during the pandemic may have been affected by changes in sexual activity associated with lockdowns.^9^ However, like reports for general ED attendance, change in presentation for UTIs may also have resulted from reductions in lower acuity attendances or patients delaying seeking healthcare. Although most UTIs are self-limiting or associated with favourable outcomes, a small number can progress to pyelonephritis requiring treatment, secondary bloodstream infection and sepsis.^10^ Delays to accessing healthcare for symptomatic UTIs during the first few years of the pandemic may have led to increased severity at presentation to hospital.

If they exist, some of these changes in patient presentation to hospital for UTIs may be signalled in national indicators of hospital prescribing, such as intravenous (IV) antibiotic use, or detectable using metrics of disease severity derived from routinely collected data.^11^ Local hospital prescribing guidelines set out when IV therapy is warranted, for example, treatment of sepsis, deep-seated infections like osteomyelitis, and patients who cannot take orally administered regimens.^12–14^ However, recommended treatment for UTIs will include the use of orally administered regimens for lower UTI, and IV administered therapy for some upper UTIs or sepsis following UTI. This means that nationally collected binary data on IV use may not be sensitive to different types of changes in disease severity at hospital admission.

The National Early Warning Score 2 (NEWS2) is a composite indicator of patient risk of decline.^15^ It is based on the sum of a scoring system of deviation from the norm across six parameters: respiration rate, oxygen saturation, systolic blood pressure, pulse rate, level of consciousness or new confusion, and temperature. The further the score from 0, the greater the deviation from the norm detected, triggering different clinical response pathways. NEWS2 benefits from good uptake across NHS hospitals. Additionally, compared with IV antibiotic use, it reflects more detailed changes in patient disease severity.

We aimed to explore whether there were changes to presentation at hospital admission for UTIs during the first 2 years of the COVID-19 pandemic. To do this, we compared two indicators of disease severity. The first was IV drug administration, which is a nationally collected indicator of prescribing generally recommended to treat more severe infection. The second was NEWS2, which is frequently collected in routine clinical care and readily available in the hospital electronic health record (EHR).

## Materials and methods

We carried out a retrospective cohort study of disease severity among patients presenting to two hospitals for UTIs, comparing those before and during the pandemic.

### Ethical approval

This research project was reviewed and approved by a Data Trust Committee as part of the PIONEER Health Data Research Hub data management process (data request reference PDR014), approved by the Health Research Authority.^16^

### Data

We analysed EHR data collected routinely through clinical care during ED and inpatient admissions using the prescribing information communication system (PICS) implemented across two hospitals, Queen Elizabeth Hospital Birmingham (QEHB) and Heartlands Hospital (HH), both part of University Hospitals Birmingham NHS Foundation Trust.

Data were extracted on the inpatient admission, including start and end datetime of hospital spells (periods of continuous care in a hospital), and episodes within each of those spells (each period of continuous care during the spell under a single nominated consultant). Linked to each admission was demographic information including sex, age on admission (aggregated to ≤17 years old, 18–25 years old, and then 10 year age bands from 26 years through to 105 years old), ethnic group (self-reported according to the NHS Data Model and Dictionary ‘ETHNIC CATEGORY’ data item),^17^ index of multiple deprivation (IMD) score (official measure of relative deprivation in England based on 39 indicators of area-level deprivation),^18^ requests and results for SARS-CoV-2 tests for infection, and ICD-10 codes.

Data on outcomes were also extracted: antibiotic administrations (drug, dose, route, datetimes of administration), and NEWS2 observations recorded during the ED attendance and admission. NEWS2 is automatically calculated by PICS, with a frequency informed by disease severity.

### Study population inclusion criteria

Between March 2020 and February 2022, England experienced the first three waves of SARS-CoV-2 infection (March–May 2020, September 2020–April 2021, and June 2021–April 2022), and three national lockdowns (26 March–23 June 2020, 5 November–2 December 2020, and 6 January–17 May 2021).^1,19^

In this study, included patients attended the ED between 1 March 2019 and 28 February 2022. The reason for attendance, recorded through the allocated Emergency Care Data Set (ECDS) code, was related to UTI or sepsis (Table S1, available as Supplementary data at *JAC-AMR* Online). ECDS codes relevant to UTI or sepsis were determined through discussion between authors, including a microbiologist at the Trust, carrying out blind review of the codes recorded for attendances during the study dates.

Patients who were then admitted to hospital had a primary ICD-10 diagnosis code indicating UTI as the reason for admission based on a code list adapted from that published by Shallcross *et al.* (Table S2).^20^ When admissions had a secondary ICD-10 diagnosis code that met this criterion (but not the primary), the primary diagnosis code underwent review to determine whether it was likely related to the UTI (and so eligible for inclusion) (Table S3).

### Study population exclusion criteria

We set out exclusion criteria to minimise the potential confounding effect of COVID-19 symptoms affecting patient need for IV antibiotic therapy or the physiological measurements taken in the NEWS2 assessment, and to prevent the inclusion of admissions where patients had developed healthcare-associated UTIs after admission. Specifically, excluded attendances occurred within 30 days of a previous hospital admission or ED attendance. Alternatively, a positive test result for SARS-CoV-2 infection was recorded either during the admission or in the 14 days preceding admission, or an ICD-10 code indicating COVID-19 was associated with the attendance/admission in diagnosis position 1 to 5.

### Study outcomes

The primary outcome of the study was the relative odds of a greater NEWS2 on admission to hospital for UTI disease, among patients before and during the first 2 years of the pandemic. The secondary outcome was the relative risk of IV antibiotic administration on admission to hospital for UTI disease, among patients before and during the first 2 years of the pandemic.

### Patient ‘time at risk’ of the study outcomes

**Figure 1.**
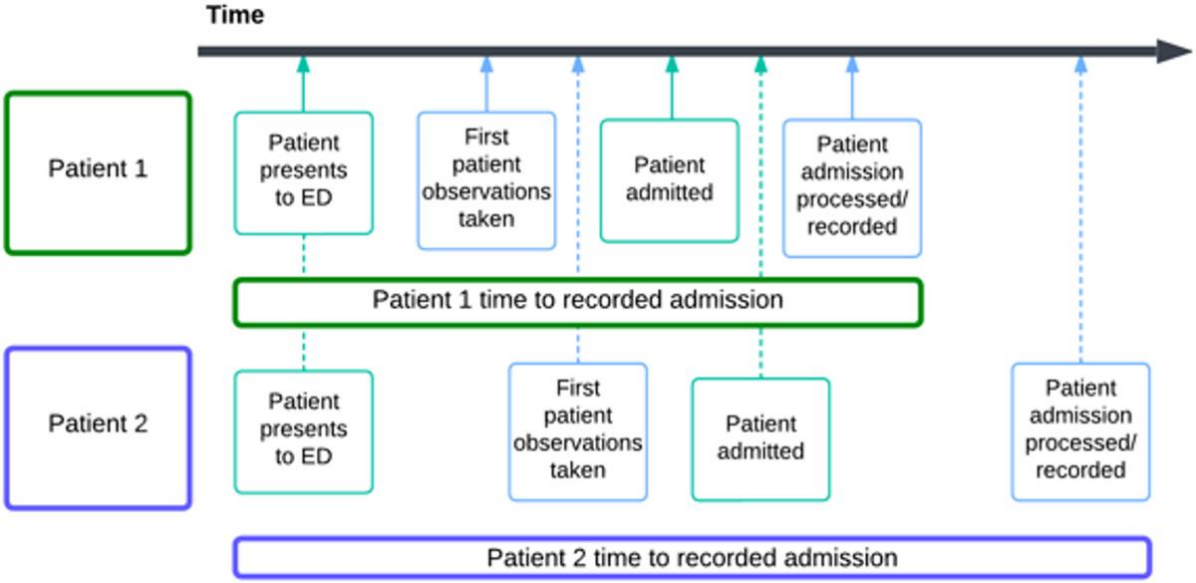
Variation in patient time from ED presentation to recorded admission datetime. Light-green boxes on the timeline indicate events that occur but are not recorded in PICS; light-blue boxes indicate events that are recorded in PICS. Solid arrow lines attached to the timeline indicate Patient 1 events; dashed arrow lines attached to the timeline indicate Patient 2 events.

Patients were recruited to the study based on admissions between 1 March 2019 and 28 February 2022, including repeat admissions. The outcomes of the study (IV antibiotic use and maximum NEWS2 on admission) were defined as those occurring up to 24 h following admission to hospital. However, the datetime of hospital admission recorded in the EHR is dependent on both when the patient was admitted and the time it takes to record that admission in hospital systems. So, patient observations or treatments can appear in the EHR before and after the recorded start of the hospital spell. This means the patient ‘time at risk’ of the outcomes for the study could be different for different patients in the cohort depending on how long it took to record the patient admission in hospital systems (Figure 1).

### Statistical analysis

First, we described the baseline characteristics (age, sex, ethnicity and IMD) of the cohort. We also described antibiotic use according to whether it was indicated for treatment of UTIs based on the Trust guidelines (Table S4). We present proportions and chi-squared tests for difference between the pre-pandemic and pandemic periods.

Next, we examined IV antibiotic administration on admission among patients presenting to hospital for UTI disease. We started by calculating the crude proportion of IV antibiotic use on admission before and during the pandemic. To estimate the relative risk of IV antibiotic administration before and during the pandemic, we developed a Poisson regression model adjusting for the potentially confounding effect of any changes in age, sex, ethnicity and IMD between periods. Age groups were aggregated to groups as described in the ‘Data’ section above. Ethnicity was categorised based on the NHS Data Model and Dictionary as described in the ‘Data’ section above. Where there was data sparsity, these variables were further aggregated to above and below 65 years old and those of a white or other ethnic group, respectively. IMD score was also aggregated to high (7–10), medium (4–6) and low (1–3) scoring patient groups. This approach to aggregation reflects differences in underlying risk of disease and management of UTIs in older versus younger adults, as well as observed differences in underlying risk of disease and healthcare seeking behaviour among patients of white versus other ethnic groups and differing socioeconomic groups.^21,22^

Next, we examined the maximum recorded NEWS2 on admission, for patients presenting to hospital for UTI disease. We started by calculating the crude median NEWS2 on admission. To estimate the OR of a higher NEWS2 during the pandemic compared with before, we developed an ordinal logistic regression model adjusting for the confounding effect of age, sex, ethnicity and IMD. Where the pandemic did not have a consistent effect on the odds of patients turning up to hospital with a higher NEWS2 (violation of the proportional odds assumption underlying ordinal logistic regression), we carried out standard logistic regression analyses comparing the relative odds of patients having a higher NEWS2 recorded on admission before and during the pandemic, i.e. comparing the odds of patients having a NEWS2 >0 on admission during and before the pandemic, then >1 on admission, and so on.

In both analyses, repeat admissions were either included in the models and adjusted for using random effects term, or, where there were very few repeat admissions which had no effect on the results of the model, they were excluded.

### Sensitivity analysis

We carried out sensitivity analyses to understand the degree to which different lengths of ‘time at risk’ between patients likely impacted the results of the study. We restricted the study outcomes to IV administration or maximum NEWS2 recorded up to 24 h following admission, and within 24 h of the first recorded NEWS2 or antibiotic drug administration. Additionally, QEHB and HH were included in the study, but HH data were only available from November 2020. This could have resulted in confounding associated with underlying differences in patient populations across sites beyond those accounted for in the models. Therefore, we also carried out sensitivity analyses excluding patients admitted to HH. Also, the youngest age group in our dataset was ≤17 years old, but NEWS2 is recommended for patients 16 years and older. In the main analysis we assumed that patients with a NEWS2 were at least 16 years old, but, given that we cannot be certain of this, we excluded these patients in sensitivity analyses. Lastly, data aggregation to binary time periods before and during the pandemic to reduce the impact of sparsity of data may have masked the effect of different waves of infection over time. Sensitivity analyses were consequently also carried out to test for differences in the effect of the pandemic by calendar year.

## Results

**Figure 2.**
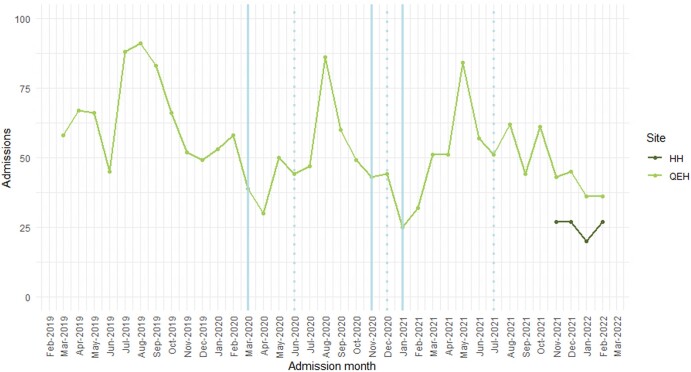
UTI presentation cohort admissions over time, by hospital. Solid blue lines indicate the start of a national lockdown, dotted blue lines indicate the phased end of a national lockdown. HH, Heartlands Hospital; QEBH, Queen Elizabeth Hospital Birmingham.

There were 1918 patients with 2047 admissions identified for inclusion in the study (Figure S1). The majority of these attendances were recorded at QEHB, with 101 recorded at HH between November 2021 and February 2022 after PICS was implemented (Figure 2). Attendances exhibited seasonal peaks between July and September in 2019 and in 2020. During 2021, the seasonal peak may have been extended between April and October and reduced in size, compared with previous years.

### IV antibiotic administration

Patients identified for inclusion in the study were in the majority female (58%), over 65 years old (65%), white (70%) and resided in more deprived areas (59% low IMD) (Table 1). There was no descriptive evidence for a difference in proportions of patients administered IV antibiotic therapy before and during the pandemic (average 68%). There was also no descriptive evidence for a difference in patterns of antibiotic use indicated for UTIs, although there may have been a slight decrease in the proportion of admissions where no antibiotic therapy was administered (17% to 15%) and a slight increase in the proportion where antibiotic therapy was indicated for minor UTIs (18% to 20%) (Table 1).

**Table 1.** Crude proportion of IV antibiotic use, and baseline characteristics of the cohort before and during the pandemic

Patient group	Pre-pandemic	Pandemic	*P* value
**Baseline characteristics**	***n* = 776 (100%)**	***n* = 1271 (100%)**	-
Administered IV antibiotics^a^	521 (67.1)	868 (68.3)	0.588
Female	471 (60.7)	712 (56.0)	0.038
≥65 years old	499 (64.3)	839 (66.0)	0.431
White ethnic group	538 (69.3)	901 (70.9)	0.454
**IMD group**	***n* = 773 (100%)**	***n* = 1271 (100%)**	0.615
Low (1–3)	465 (60.2)	750 (59.0)	
Middle (4–6)	211 (27.3)	342 (26.9)	
High (7–10)	97 (12.5)	179 (14.1)	
**UTI indication for drug use^b^**	***n* = 776 (100%)**	***n* = 1271 (100%)**	0.036
No antibiotic use	134 (17.3)	188 (14.8)	
Minor UTI	136 (17.5)	254 (20.0)	
Severe UTI	500 (64.4)	803 (63.2)	
Not indicated for UTI	6 (0.8)	26 (2.0)	

^a^Within 24 h of admission.

^b^Classification of antibiotic use by UTI-related indication for use based on Trust guidelines (Table S4).

The limited apparent effect of the pandemic on IV antibiotic use persisted after adjusting for age, sex, ethnicity and IMD [adjusted risk ratio (aRR): 1.02; 95% confidence interval (CI): 0.91–1.15] (Table 2).

**Table 2.** Unadjusted and adjusted associations with IV antibiotic use^a^

Patient group comparison	RR IV antibiotic administration (95% CI)	aRR IV antibiotic administration (95% CI)
During the pandemic versus before	1.02 (0.91–1.13)	1.02 (0.91–1.15)
Female versus male	**0.83 (0.75–0.93)**	**0.83 (0.74–0.93)**
65+ versus ≤65 years old	1.02 (0.91–1.14)	0.98 (0.86–1.09)
Other ethnic groups versus white ethnicities	0.98 (0.87–1.10)	0.98 (0.86–1.10)
Middle IMD (4–6) versus low (1–3)	1.06 (0.94–1.20)	1.06 (0.93–1.20)
High IMD (7–10) versus low (1–3)	1.03 (0.88–1.21)	1.04 (0.88–1.23)

^a^Risk ratio (RR) and risk ratio adjusted for all variables listed in the table (aRR) shown with 95% confidence intervals (CI). Bold text indicates *P* values <0.05.

### NEWS2

**Figure 3.**
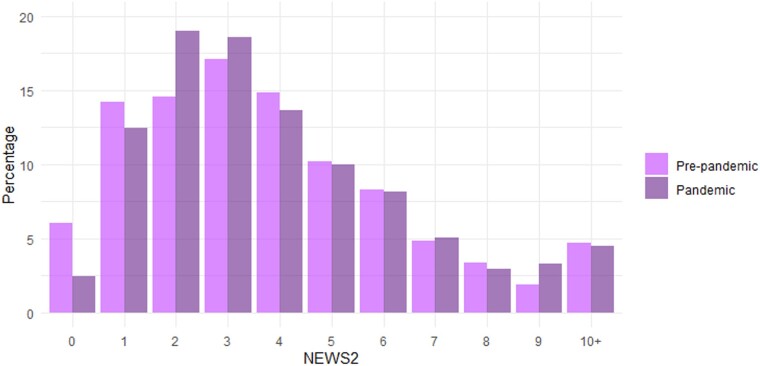
Proportion of presentations by maximum NEWS2 recorded within 24 h of admission, before and during the pandemic.

Of those 2047 admissions eligible for inclusion in this analysis, 67 did not have a NEWS2 recorded up to 24 h following admission. Patients with a NEWS2 recorded had an age, sex, ethnicity and IMD demographic profile similar to the entire cohort included in the analysis of IV drug administration: 58% were female, 66% were 65 years or older, 71% were of a white ethnic group, and 59% resided in a low IMD scoring area (Table S5). Very few repeat admissions across outcome groups meant that only first presentation to hospital was included the main analysis. The median NEWS2 on admission in the cohort was the same in the period before and during the pandemic, i.e. 3 (IQR: 2–5), but descriptive analyses suggested a decrease in presentation with a lower NEWS2 (0 or 1) and more presentation with a NEWS2 of 2 during the pandemic (Figure 3). However, presentations with a NEWS2 >2 appeared similar across both time periods.

Through standard logistic regression analysis we saw the descriptive findings of Figure 3 persist after adjusting for age, sex, ethnicity and IMD group (Table 3). Specifically presentation with a NEWS2 of 0 was less likely during the pandemic [adjusted odds ratio (aOR): 0.37; 95% CI: 0.25–0.65], and presentation with a NEWS2 >0 or >1 was more likely (aOR: 2.74; 95% CI: 1.54–4.02, and aOR: 1.52; 95% CI: 1.18–1.94, respectively). This increase in severity of disease was limited to patient groups presenting with a lower NEWS2, i.e. increased score 2 relative to 0 and 1. Similar proportions of patients with a NEWS2 of 3 or more on admission presented to hospital before and during the pandemic (aOR: 1.06; 95% CI: 0.87–1.29), as well as similar proportions of patients with a NEWS2 >4 and >6 at hospital presentation (aOR: 1.07; 95% CI: 0.87–1.31, and aOR: 1.03; 95% CI: 0.79–1.34, respectively).

**Table 3.** Adjusted associations between the pandemic and NEWS2 on admission^a^

Patient group comparison	aOR NEWS2 >0	aOR NEWS2 >1	aOR NEWS2 >2	aOR NEWS2 >4	aOR NEWS2 >6
During the pandemic versus before	**2.74 (1.54–4.02)**	**1.52 (1.18–1.94)**	1.06 (0.87–1.29)	1.07 (0.87–1.31)	1.03 (0.79–1.34)
Female versus male	1.03 (0.62–1.68)	0.80 (0.62–1.04)	0.87 (0.71–1.06)	0.90 (0.74–1.10)	**0.76 (0.58–0.98)**
65+ versus ≤65 years old	**3.00 (1.78–5.00)**	**2.06 (1.58–2.67)**	**1.81 (1.47–2.23)**	**1.60 (1.28–2.00)**	**1.37 (1.02–1.85)**
Other ethnic groups versus white ethnicities	0.67 (0.41–1.11)	**0.69 (0.53–0.90)**	**0.78 (0.63–0.96)**	**0.78 (0.62–0.98)**	**0.62 (0.45–0.85)**
Middle IMD (4–6) versus low (1–3)	1.33 (0.74–2.56)	1.08 (0.80–1.46)	1.19 (0.94–1.50)	1.24 (0.99–1.55)	**1.44 (1.09–1.91)**
High IMD (7–10) versus low (1–3)	0.96 (0.49–2.05)	0.86 (0.60–1.25)	0.93 (0.69–1.25)	0.97 (0.72–1.31)	0.94 (0.62–1.40)

^a^ORs adjusted for all variables in the table presented (aOR) with 95% confidence intervals. Bold text indicates *P* values <0.05.

### Sensitivity analyses

Sensitivity analyses provided no evidence for an effect of patient time at risk, inclusion of data from patients ≤17 years old, or HH on the results of the main analysis (Tables S6–S8). Comparing UTI presentation in 2019 (pre-pandemic) with that separately in 2021 and 2020 (during the pandemic), indicated there was still no observable effect of the pandemic on rates of IV antibiotic administration when analysed by year (Table S6). For NEWS2, small increases in score at hospital presentation during the pandemic persisted in this calendar year sensitivity analysis; however, it revealed that increases in disease severity at hospital presentation were likely concentrated in 2021 (the second year of the pandemic) (aOR of NEWS2 >1: 1.80; 95% CI: 1.34–2.43), and that they were unlikely to have been present among hospital admissions in 2020 (aOR of NEWS2 >1: 1.24; 95% CI: 0.92–1.68) (Table S7).

## Discussion

In this study, we explored whether during the pandemic there were changes to the way that patients presented to hospital for UTIs. We applied two indicators of disease severity at the point of hospital presentation: IV antibiotic use (a national indicator of prescribing) and NEWS2 (a widely adopted indicator of risk of patient decline prospectively recorded in the EHR as part of clinical care). During the pandemic, patients were more likely to present to hospital with a NEWS2 of 2 but less likely with a score of 0 or 1. This limited increase in disease severity during the pandemic did not appear to result in changes to IV antibiotic usage (aRR: 1.02; 95% CI: 0.91–1.15). Applying IV antibiotic usage as a proxy indicator of severity of disease presentation, available through nationally reported indicators of antibiotic prescribing based on pharmacy stock data, may not detect milder changes in levels of disease severity. Simple indicators available in routinely collected EHR data could provide useful and potentially timely additional insights into changes in prescriber behaviour.

These findings seem broadly concordant with a limited number of existing studies that also evaluate a similar outcome. Patients included in this study presenting to hospital for a UTI had a median NEWS2 on admission of 3 (IQR: 2–5). This is similar to a study of patients at Oxford University Hospitals Trust, which reported a mean NEWS2 of the same during May 2019—August 2021.^23^ The slight increase in disease severity at hospital presentation observed in our study during the pandemic may reflect patients responding to public messaging to ‘stay home, and protect the NHS’, resulting in some delays to seeking care and slightly more severe disease presentations to hospital. However, given the results of sensitivity analyses suggesting that this change was driven by differences in presentation in the second year of the pandemic (not the first), it may more likely represent a change in patient population due to a series of other behavioural and environmental changes during the first 2 years of the pandemic.

A limited increase in disease severity (denoted by NEWS2) on admission is also concordant with our second finding of no observable change in IV antibiotic use on admission for UTI during the pandemic. IV antibiotic therapy intervention is unlikely to be warranted for those patients with milder illness (NEWS2 of 1 or 2 on admission). Even for severe infections, IV therapy is usually only indicated where high serum concentrations need to be rapidly and reliably achieved. A study of hospital antibiotic prescribing in Scotland, also based on electronic prescribing and medicines administration data, did not find any variation in duration of IV antibiotic prescriptions associated with the pandemic.^24^ Previously observed increases in hospital antibiotic use may instead have been driven by changes in the use of orally administered therapy, prescribing during the admission, and/or use for patients being treated for non-UTIs.^11^

Routine clinical data increasingly collected through hospital EHRs likely represent a potential opportunity to increase nuance in our understanding of patterns of prescribing and stewardship. However, further research is needed to improve the approach and consistency with which these data are exploited.

### Limitations

This study has a number of limitations. Firstly, sparsity of admissions resulting in the need to aggregate time periods to ‘before’ and ‘during’ the pandemic may mask variation in outcomes between individual years or months. Similarly, aggregation of ethnic groups, IMD and age for the main analysis does not fully represent more complex patterns of epidemiology. Sensitivity analyses suggested this may have affected analyses of NEWS2 on presentation to hospital, but not analyses of IV antibiotic administration. Secondly, the data included in this study were largely based on a single site, limiting the extent to which the findings of this research are nationally generalizable. Thirdly, this study focused exclusively on patient engagement with hospital care services and includes consideration of a relatively narrow set of potentially confounding variables. Factors such as engagement with primary care and multimorbidity may also influence the results of the study.

### Conclusions

In this study, we did not find evidence that IV antibiotic use on admission for UTIs changed during the pandemic. However, our findings do suggest that there may have been a small increase in disease severity at hospital presentation. Further research is required to validate these findings and understand whether routine data could support a more nuanced understanding of local antimicrobial prescribing practices.

## Supplementary Material

dlae067_Supplementary_Data

## Supplementary data

Figure S1 and Tables S1 to S8 are available as Supplementary data at *JAC-AMR* Online.
