# Supplementary material for: Comparing indicators of disease severity among patients presenting to hospital for urinary tract infections before and during the COVID-19 pandemic
Source: JAC Antimicrob Resist. 2024 Apr 24;6(2):dlae067. doi: 10.1093/jacamr/dlae067 (PMC11040270; doi:10.1093/jacamr/dlae067)
Supplement: dlae067_Supplementary_Data [file dlae067_supplementary_data.docx]

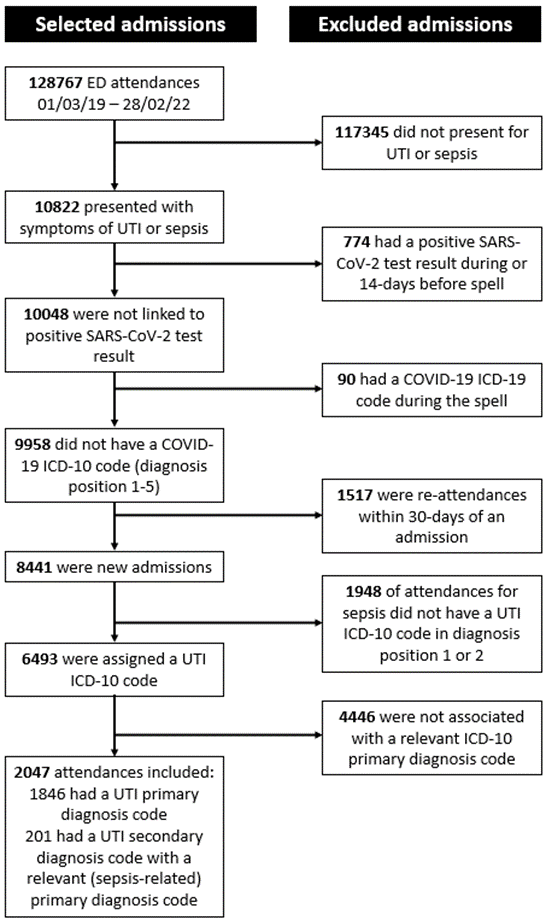


**Figure S 1 Flow diagram selecting hospital admissions for inclusion in analyses of UTI disease presentation**

**Table S 1 Emergency care data set (ECDS) codes for potential UTI**

Determined through discussion between authors carrying out a blind review of ECDS codes attached to ED attendances during the study period.

| **ECDS code** | **ECDS code description** |
| --- | --- |
| 1418111000 | Pyelonephritis |
| 1513111000 | Urinary tract infection |
| 1514111000 | Sepsis |
| 1911125000 | Delirium (acute confusion) |
| 1418121000 | Acute renal failure |
| 1613119000 | Haematuria |
| 1415121000 | Neutropaenic sepsis |

**Table S 2 Primary or secondary international classification of diseases (ICD) 10th revision codes for UTI**

Adapted from a list published by Shallcross et al. (2020). Admissions allocated primary ICD-10 codes from this list were eligible for inclusion in the study. Those with a secondary ICD-10 code from this list were eligible for inclusion only if the primary ICD-10 code was deemed likely relevant to the secondary UTI code (based on microbiologist review).

| **ICD-10 code** | **ICD-10 code description** |
| --- | --- |
| N10 | Acute tubulo-interstitial nephritis |
| N11 | Chronic tubulo-interstitial nephritis |
| N12 | Tubulo-interstitial nephritis not specified as acute or chronic |
| N136 | Pyonephrosis |
| N159 | Renal tubulo-interstitial disease, unspecified |
| N390 | Urinary tract infection, site not specified |
| N300 | Acute cystitis |
| N308 | Other cystitis |
| N309 | Cystitis, unspecified |

**Table S 3 Primary international classification of diseases (ICD) 10th revision codes deemed likely linked to a secondary diagnosis code for UTI**

Determined through discussion between authors carrying out review of primary diagnosis codes allocated to those admissions with a secondary diagnosis code for UTI as defined in Table S2.

| **ICD-10 code** | **ICD-10 code description** |
| --- | --- |
| R41 | Disorientation |
| N10 | Acute tubulonephritis |
| H81 | Vestibular function disorder |
| R11 | Nausea and vomiting |
| R10 | Abdominal and pelvic pain |
| R31 | Haematuria |
| N17 | Acute renal failure |
| M10 | Gout |
| M54 | Dorsalgia |
| N39 | Urinary system disorder |
| F05 | Delirium |
| R33 | Urine retention |
| N13 | Obstructive and reflux uropathy |
| N12 | Tubulo-interstitial nephritis |
| A41 | Sepsis |

**Table S 4 UTI-related indication for antibiotic drug use on admission, based on Trust guidelines**

| **Drug** | **Route** | **Indication for UTI** |
| --- | --- | --- |
| Aciclovir | Oral | Not indicated for UTI |
| Amoxicillin | IV | Severe UTI |
| Amoxicillin | Oral | Minor UTI |
| Augmentin | IV | Severe UTI |
| Augmentin | Oral | Minor UTI |
| Ceftazidime | IV | Not indicated for UTI |
| Ceftriaxone | IV | Severe UTI |
| Cefuroxime | IV | Not indicated for UTI |
| Cefalexin | Oral | Minor UTI |
| Ciprofloxacin | IV | Severe UTI |
| Ciprofloxacin | Oral | Minor UTI |
| Chloramphenicol | Eye | Not indicated for UTI |
| Chloramphenicol | IV | Not indicated for UTI |
| Clarithromycin | IV | Not indicated for UTI |
| Clarithromycin | Oral | Not indicated for UTI |
| Clindamycin | IV | Not indicated for UTI |
| Doxycycline | Oral | Not indicated for UTI |
| Ertapenem | IV | Severe UTI |
| Flucloxacillin | IV | Not indicated for UTI |
| Flucloxacillin | Oral | Not indicated for UTI |
| Fluconazole | Oral | Not indicated for UTI |
| Fosfomycin | IV | Severe UTI |
| Fosfomycin | Oral | Minor UTI |
| Gentamicin | IV | Severe UTI |
| Levofloxacin | IV | Severe UTI |
| Levofloxacin | Oral | Minor UTI |
| Linezolid | Oral | Not indicated for UTI |
| Meropenem | IV | Severe UTI |
| Mupirocin | Nasal | Not indicated for UTI |
| Nitrofurantoin | Oral | Minor UTI |
| Piperacillin-tazobactam | IV | Severe UTI |
| Trimethoprim | Oral | Minor UTI |
| Vancomycin | IV | Not indicated for UTI |

**Table S 5 Baseline characteristics of all eligible admissions (included in the analysis of IV drug administration rate), compared to those with a recorded NEWS2**

| **Variable** | **Entire Cohort**  **(N=2047)** | **NEWS2 cohort**  **(N=1980)** | **p-value** |
| --- | --- | --- | --- |
| **Sex** | | | 0.915^a^ |
| Male | 864 (42.2%) | 839 (42.4%) |  |
| Female | 1183 (57.8%) | 1141 (57.6%) |  |
| **Age on admission** | | | 0.692^b^ |
| <=17 | 14 (0.7%) | 11 (0.6%) |  |
| 18 - 24 | 100 (4.9%) | 95 (4.8%) |  |
| 25 - 34 | 141 (6.9%) | 133 (6.7%) |  |
| 35 - 44 | 100 (4.9%) | 93 (4.7%) |  |
| 45 - 54 | 158 (7.7%) | 154 (7.8%) |  |
| 55 - 64 | 196 (9.6%) | 192 (9.7%) |  |
| 65 - 74 | 294 (14.4%) | 286 (14.4%) |  |
| 75 - 84 | 488 (23.8%) | 474 (23.9%) |  |
| 85 - 94 | 494 (24.1%) | 482 (24.3%) |  |
| 95 - 104 | 62 (3.0%) | 60 (3.0%) |  |
| **Ethnic group** | | | 0.998^a^ |
| Asian | 278 (13.6%) | 264 (13.3%) |  |
| Black | 75 (3.7%) | 68 (3.4%) |  |
| Mixed | 19 (0.9%) | 19 (1.0%) |  |
| Not stated | 188 (9.2%) | 179 (9.0%) |  |
| Other | 48 (2.3%) | 48 (2.4%) |  |
| White | 1439 (70.3%) | 1402 (70.8%) |  |
| **IMD** | | | 1.000^a^ |
| Missing | 3 | 3 |  |
| 1 | 611 (29.9%) | 591 (29.9%) |  |
| 2 | 336 (16.4%) | 326 (16.5%) |  |
| 3 | 268 (13.1%) | 257 (13.0%) |  |
| 4 | 174 (8.5%) | 167 (8.4%) |  |
| 5 | 239 (11.7%) | 231 (11.7%) |  |
| 6 | 140 (6.8%) | 137 (6.9%) |  |
| 7 | 125 (6.1%) | 122 (6.2%) |  |
| 8 | 63 (3.1%) | 62 (3.1%) |  |
| 9 | 47 (2.3%) | 45 (2.3%) |  |
| 10 | 41 (2.0%) | 39 (2.0%) |  |

1. Pearson’s Chi-squared test
2. Trend test for ordinal variables

**Table S 6 Sensitivity analyses: Adjusted associations between the pandemic and IV antibiotic administration on admission**

| **Patient group** | **Included cohort in the main analysis (%)** | **aRR (95% CI) – main analysis** | **aRR (95% CI) – restricting patient time at risk** | **aRR (95% CI) – comparing years** | **Included cohort excluding HH site (%)** | **aRR (95% CI) – excluding HH site** |
| --- | --- | --- | --- | --- | --- | --- |
| During the pandemic versus pre-pandemic | 1192 (62) | 1.02 (0.91 – 1.15) | 1.02 (0.91 – 1.15) | - | 1170 (60) | 1.03 (0.92 – 1.16) |
| Year 2020 versus 2019 | 499 (26) | - | - | 1.02 (0.89 – 1.18) | - | - |
| Year 2021 versus 2019 | 693 (36) | - | - | 1.03 (0.90 – 1.17) | - | - |
| Female versus male | 1112 (58) | **0.83 (0.74 – 0.93)** | **0.82 (0.74 – 0.92)** | **0.83 (0.74 – 0.93)** | 1052 (58) | **0.83 (0.74 – 0.93)** |
| 65+ years old versus <65 years | 1250 (65) | 0.98 (0.86 – 1.09) | 0.96 (0.85 – 1.08) | 0.96 (0.86 – 1.09) | 1182 (65) | 0.96 (0.85 – 1.08) |
| Other ethnic groups versus white ethnicities | 574 (30) | 0.98 (0.86 – 1.10) | 0.98 (0.86 – 1.11) | 0.98 (0.86 – 1.10) | 532 (29) | 0.98 (0.86 – 1.11) |
| Middle IMD (4 – 6) versus low (1-3) | 517 (27) | 1.06 (0.93 – 1.20) | 1.05 (0.93 – 1.19) | 1.06 (0.93 – 1.20) | 502 (28) | 1.06 (0.93 – 1.21) |
| High IMD (7 – 10) versus low (1-3) | 256 (13) | 1.04 (0.88 – 1.23) | 1.04 (0.88 – 1.23) | 1.04 (0.88 – 1.23) | 235 (13) | 1.06 (0.89 – 1.25) |

Relative rate adjusted for all variables listed in the table (aRR) shown. **Bold** text indicates p-values <0.05.

**Table S 7 Sensitivity analyses: Adjusted associations between the pandemic and patients presenting to hospital with a NEWS2 > 1**

Odds ratios adjusted for all variables listed in the table (aOR) shown. **Bold** text indicates p-values <0.05.

| **Patient group** | **Included cohort in main analysis (%)** | **aOR NEWS2 >1 (95% CI) - main analysis** | **aOR NEWS2 >1 (95% CI) – restricting patient time at risk** | **aOR NEWS2 >1 (95% CI) – comparing years** | **Included cohort excluding HH site (%)** | **aOR NEWS2 >1 (95% CI) – excluding HH site** | **Included cohort excluding <=17 year olds** | **aOR NEWS2 >1 (95% CI) – excluding <=17 year olds** |
| --- | --- | --- | --- | --- | --- | --- | --- | --- |
| During the pandemic versus pre-pandemic | 1157 (62) | **1.52 (1.18 – 1.95)** | **1.48 (1.16 – 1.88)** | - | 1076 (61) | **1.51 (1.17 – 1.95)** | **1151 (62)** | **1.52 (1.18 – 1.95)** |
| Year 2020 versus 2019 | 496 (27) | **-** | **-** | 1.24 (0.92 – 1.68) | - | **-** | **-** | **-** |
| Year 2021 versus 2019 | 661 (36) | **-** | **-** | **1.80 (1.34 – 2.43)** | - | **-** | **-** | **-** |
| Female versus male | 1074 (58) | 0.80 (0.62 – 1.04) | 0.81 (0.63 – 1.04) | 0.80 (0.61 – 1.04) | 1027 (58) | 0.84 (0.64 – 1.09) | 1069  (58) | 0.81 (0.62 – 1.05) |
| 65+ years old versus <65 years | 1215 (66) | **2.06 (1.58 – 2.67)** | **1.90 (1.48 – 2.45)** | **2.06 (1.59 – 2.67)** | 1158 (65) | **2.14 (1.64 – 2.79)** | 1217 (66) | **2.08 (1.60 -2.70)** |
| Other ethnic groups versus white ethnicities | 547 (29) | **0.69 (0.53 – 0.90)** | **0.69 (0.54 – 0.90)** | **0.69 (0.53 – 0.90)** | 518 (29) | **0.72 (0.55 – 0.94)** | **544 (30)** | **0.70 (0.54 – 0.92)** |
| Middle IMD (4 – 6) versus low (1-3) | 502 (27) | 1.08 (0.80 – 1.46) | 1.01 (0.76 – 1.35) | 1.09 (0.81 – 1.47) | 491 (28) | 1.08 (0.59 – 1.26) | 500 (27) | 1.07 (0.80 – 1.45) |
| High IMD (7 – 10) versus low (1-3) | 248 (13) | 0.86 (0.60 – 1.25) | 0.90 (0.63 – 1.30) | 0.87 (0.60 – 1.26) | 232 (13) | 0.86 (0.59 – 1.26) | 247 (13) | 0.85 (0.59 – 1.24) |

**Table S 8 Sensitivity analyses: Adjusted associations between the pandemic and patients presenting to hospital with a NEWS2 > 4**

| **Patient group comparisons** | **Included cohort in main analysis (%)** | **aOR >4 (95% CI)** **– main analysis** | **aOR >4 (95% CI) – restricting patient time at risk** | **aOR NEWS2 >4 (95% CI) – comparing years** | **Included cohort excluding HH site (%)** | **aOR NEWS2 >4 (95% CI) – excluding HH site** | **Included cohort excluding <=17 year olds** | **aOR NEWS2 >4 (95% CI) – excluding <=17 year olds** |
| --- | --- | --- | --- | --- | --- | --- | --- | --- |
| During the pandemic versus pre-pandemic | 1157 (62) | 1.06 (0.87 – 1.29) | 1.08 (0.88 - 1.32) | - | 1076 (61) | 1.05 (0.86 - 1.29) | **1151 (62)** | 1.06 (0.87 – 1.30) |
| Year 2020 versus 2019 | 496 (27) | - | - | 1.15 (0.90 – 1.48) | - | - | **-** | - |
| Year 2021 versus 2019 | 661 (36) | - | - | 1.00 (0.80 – 1.26) | - | - | **-** | - |
| Female versus male | 1074 (58) | 0.87 (0.71 – 1.06) | 0.92 (0.75 - 1.13) | 0.91 (0.74 – 1.11) | 1027 (58) | 0.90 (0.73 - 1.10) | 1069  (58) | 0.91 (0.74 – 1.11) |
| 65+ years old versus <65 years | 1215 (66) | **1.81 (1.47 – 2.23)** | **1.59 (1.27 - 1.99)** | **1.60 (1.28 – 2.00)** | 1158 (65) | **1.62 (1.29 - 2.04)** | 1217 (66) | **1.60 (1.29 – 2.00)** |
| Other ethnic groups versus white ethnicities | 547 (29) | **0.78 (0.63 – 0.96)** | **0.77 (0.61 - 0.97)** | **0.78 (0.62 – 0.98)** | 518 (29) | **0.78 (0.62 - 0.99)** | **544 (30)** | **0.80 (0.63 – 1.00)** |
| Middle IMD (4 – 6) versus low (1-3) | 502 (27) | 1.19 (0.94 – 1.50) | 1.24 (0.99 - 1.55) | 1.24 (0.99 – 1.55) | 491 (28) | **1.25 (1.00 - 1.57)** | 500 (27) | 1.24 (0.99 – 1.55) |
| High IMD (7 – 10) versus low (1-3) | 248 (13) | 0.93 (0.69 – 1.25) | 0.94 (0.69 - 1.27) | 0.97 (0.72 – 1.30) | 232 (13) | 0.95 (0.69 - 1.29) | 247 (13) | 0.98 (0.72 – 1.32) |

Odds ratios adjusted for all variables listed in the table (aOR) shown. **Bold** text indicates p-values <0.05.
